# Targeted Intracellular Heat Transfer in Cancer Therapy: Assessment of Asparagine-laminated Gold Nanoparticles in Cell Model of T cell Leukemia

**Published:** 2017-03

**Authors:** Shadab SHAHRIARI, Maryam BAKHSHI, Ahmad Reza SHAHVERDI, Azar BERAHMEH, Farnaz SAFAVIFAR, Mohammad Reza KHORRAMIZADEH

**Affiliations:** 1. Biosensor Research Center, Endocrinology and Metabolism Cellular-Molecular Sciences Institute, Tehran University of Medical Sciences, Tehran, Iran; 2. Biotechnology Research Center, School of Pharmacy, Tehran University of Medical Sciences, Tehran, Iran; 3. Dept. of Pathobiology, School of Public Health, Tehran University of Medical Sciences, Tehran, Iran; 4. Endocrinology and Metabolism Research Center, Endocrinology and Metabolism Clinical Sciences Institute, Tehran University of Medical Sciences, Tehran, Iran

**Keywords:** Gold nanoparticles, Asparagine, Acute lymphoblastic leukemia, CCRF-CEM cells

## Abstract

**Background:**

High temperatures have destructive effects on cancer cells by damaging proteins and structures within cells. Gold nanoparticles (AuNPs) can act as drug delivery vehicles, especially for cancer therapy. Due to the selective intake of asparagine molecules into malignant cells, AuNPs were coated with asparagine; and CCRF-CEM human T-cell leukemia cells were treated with the new combination, Asn-AuNPs, at 39 °C.

**Methods:**

The co-authors from a number of collaborative labs located at Tehran University of Medical Sciences, Tehran, Iran, have initiated the idea and preliminary design of this study in 2011. Hydroxyl surfaced AuNPs were preliminary prepared by tannin free ethanol extract of black tea leaves. These biogenic AuNPs were further capped with asparagines to form asparagine-gold nanoparticle conjugates (Asn-AuNP conjugates). Then CCRF-CEM human T-cell leukemia cells were separately treated with different concentrations of AuNPs and Asn-AuNP conjugates (3, 30, 300 μg/mL). MTT assay and zymography analysis were carried out, and the apoptotic and necrotic effects of Asn-AuNPs were determined in comparison with AuNPs, using flow cytometry assay.

**Results:**

Asn-AuNP conjugates at 300 μg/mL significantly inhibited MMPs at 39 °C, compared to AuNPs. In terms of cytotoxicity, a remarkable decrease was observed in the percentage of viable cells treated with Asn-AuNP conjugates, rather than AuNPs. Moreover, the AuNPs and Asn-AuNP conjugates enhanced the level of apoptosis at almost similar rates.

**Conclusion:**

AuNPs are coated with asparagine molecules and the temperature is slightly increased by 2 °C, the apoptosis is not only enhanced among cells but also shifts to necrosis in higher concentrations of Asn-AuNP conjugates. More investigations should be carried out to explain the exact mechanism underlying the necrotic effects of Asn-AuNPs.

## Introduction

Gold nanomaterials have wide applications in nanomedicine, particularly in imaging and therapy of cancers. Gold nanorods have been applied in molecular imaging, gold nanoshells, and gold nanocages in drug delivery systems (1, 2). Gold nanoparticles (AuNPs) can act as biosensors, cytotoxic agents, and drug delivery vehicles (3–5). AuNPs have been shown to enhance the antibacterial effect of a number of antibiotics against bacterial cells (6). A number of factors make AuNPs desirable candidates for treating cancer cells: (i) Gold nanoparticles have unique optical, physical and chemical properties due to their size (<100 nm) and shape; (ii) They have high surface area which provides dense drug loading; (iii) These particles are biocompatible and are readily available for conjugation with small biomolecules; (iv) Due to small size and uniform dispersion they can easily reach to the targeted site with blood flow; (v) They are non-cytotoxic to the normal cells; and (vi) Gold nanoparticles are easily synthesized by various methods (7, 8). In addition, to their ability to penetrate deep into tissues or even into cells, the unique optical and magnetic properties of gold nanocomposites make it possible to track their intracellular trafficking and localization (9).

Hyperthermia is defined as supra-normal body temperatures. During treatment, the body temperature reaches a level between 39.5 °C and 40.5 °C (10). However, other researchers define hyperthermia between 41.8 °C–42 °C to near 43 °C–44 °C in USA and Russia, respectively (11). Temperature and time are interrelated, with longer times at temperature meaning more cancer cell kill but also higher risk of toxicity. Hyperthermia may kill or weaken tumor cells, and is controlled to limit effects on healthy cells. Therefore, high temperature may cause cancerous cells to undergo apoptosis in direct response to applied heat, while healthy tissues can more easily maintain a normal temperature. It may also be used in combination with other forms of cancer therapy, such as radiation therapy and chemotherapy (12). This combination therapy for cancer cells is particularly effective since high temperature makes cancer cells more sensitive to radiation or may also enhance the effects of certain anticancer drugs (13). In this work, the temperature used in combination with the gold nanomaterials was 39 °C, which is within the range of thermal tolerance and could be easily obtained by inducing a fever.

Tumor cells selectively intake asparagine molecules (the amino acids necessary for malignant cells to proliferate) (14). A common medication commonly used for treating acute lymphoblastic leukemia (ALL) in children is L-asparaginase, an enzyme that inhibits protein synthesis by depleting the sources of the asparagine molecules (15). The concept of this work was the hypothesis that, due to the selective intake of asparagine molecules into malignant cells (14), asparagine-gold nanoparticle conjugates (Asn-AuNP conjugates) might have enhanced cytotoxic and apoptotic properties, compared to free AuNPs.

Many efforts have been made to enhance the efficacy of gold nanomaterials against cancer cells. For instance, the apoptotic effect of AuNPs in B-chronic lymphocytic leukemia cells increases when the AuNPs are coated with anti-vascular endothelial growth factor (VEGF) antibodies (16). In this work, a new strategy was designed to enhance the apoptotic and cytotoxic effects of AuNPs. AuNPs were coated with asparagine, and *CCRF-CEM* human T-cell leukemia cells were treated with the new combination at 39 °C. Finally, the cytotoxic, apoptotic, and necrotic effects of the Asn-AuNP conjugates were evaluated in comparison with asparagine free AuNPs. The underlying concept was that asparagine molecules on the surface of Asn-AuNP conjugates might facilitate the selective intake of nanoparticles into malignant cells, thereby improving the quality of hyperthermia. The cytotoxicity and matrix metalloproteinase (MMP) inhibitory activity of AuNPs increases when they are coated with asparagine molecules. Moreover, we observed that coating nanoparticles with asparagine resulted in less apoptosis and more necrosis among CCRF-CEM cells.

## Materials and Methods

### Synthesis and characterization of AuNPs

The idea and preliminary design of this study was triggered in 2011. Based on the paucity of experimental records and accessibility assessments, numerous methodological modifications and work setups were done by the inter-departmental collaboration at several lab-settings located in Tehran University of Medical Sciences to implement the research and achieve the optimal outcomes. The reduction of the aqueous solution of chloroauric acid (HAuCl_4_) is a widely used method for synthesizing gold colloids (17, 18). In this work, the tannin free ethanol extract was used for green synthesis of AuNPs based on a protocol (19). In brief, the black tea leaves of *Camellia sinensis* were pulverized, and the ethanol extract was prepared by macerating the powder for 48 h at room temperature. Tannins were absorbed from the collected ethanol extract by the Sephadex LH 20 beads, and the filtrate was evaporated to yield brownish, viscous residues. A stock solution was then prepared in ethanol (10 mg/mL) and reserved at 4 °C for the next experiments. An aqueous chloroauric acid solution (1 mM) was added separately to the reaction vessel containing the tannin free ethanol extract of black tea fraction (10% v/v), and the resulting mixture was allowed to stand for 15 min at room temperature (25 °C). The reduction of the Au^+3^ ions by this ethanol extract in the solution was monitored after 15 min by sampling 2 mL of the aqueous component and measuring the UV–visible spectrum of the solutions. The sample was diluted three times with distilled water, and the UV–visible spectra of these samples were measured on a Labomed Model UVD-2950 UV-VIS Double Beam PC Scanning spectrophotometer, operated at a resolution of 2 nm. The prepared colloid was further characterized with transmission electron microscopy (TEM 208 Philips) and energy-dispersive spectroscopy (EDS) (EM 208 Philips).

### Preparation of Asn-AuNP conjugates and characterization

The 20 mg/mL solution of L-asparagine was added to the AuNPs colloid (10 mg/mL), and the mixture was incubated at 25 °C in a shaker incubator (200 rpm) for 24 h. The resultant nanoparticles were centrifuged (20000 gr) and washed three times with highly double-distilled water. The surface chemistry of the washed AuNPs was studied using Fourier transform infrared spectroscopy (FTIR) (Nicolet Magna 550, *Madison, WI, USA*). The FTIR spectra of the washed free AuNPs and asparagine powder were also recorded and compared with the FTIR spectrum of the Asn-AuNP conjugates. In addition, the thermal decomposition characteristics of Asn-AuNPs were determined using Thermogravimetric TGA Q50 V6.3 Analyzer equipment.

### Cell culture

The human leukemic lymphoblasts (CCRF-CEM cell line) were purchased from the National Cell Bank of Iran (Pasteur Institute of Iran, Tehran, Iran). Cells were maintained in DMEM medium supplemented with 10% fetal calf serum, 100 U/mL penicillin, 100 μg/mL streptomycin, and 5% CO_2_, at 37 °C and saturated humidity. Cells were seeded at an initial density of 10000 cells/well in 96-well tissue culture plate. In the test group, cells were treated with increasing concentrations of 3, 30, and 300 μg/mL solutions of the Asn-AuNP conjugates, while the control group received concentrations of 3, 30, and 300 μg/mL of AuNPs. All experiments were carried out six times.

### MTT cytotoxicity assay and Zymography analysis

Cells were cultured in the logarithmic phase of growth, and the cytotoxic MTT assay was carried out according to Freshney’s instructions (20), by preliminarily seeding 50000 cancer cell, in triplicates, in a 100 μL growth medium in the presence of increasing concentrations of AuNPs and Asn-AuNP conjugates. Different concentrations of 3, 30, and 300 μg/mL of the AuNPs and Asn-AuNP conjugates were prepared by diluting the stock solution of these compounds with complete culture medium. Samples were incubated in 96-well plates individually, with subsequent incubation of cells at 39 °C in 5% CO_2_ for 48 h. At this stage, 50 μL was removed from each sample and transferred to separate microtubes for further zymography analysis. Cells were thereafter treated with 50 μL of the MTT solution and incubated at 37 °C for 4 h. After formazan crystals were dissolved in 0.04 N HCl in isopropanol, the density of the developed purple color was read at 580 nm using a conventional ELISA reader instrument (Dynatech Laboratories, Inc, United States). An MTT assay was performed in six replicates for each experiment. Cytotoxicity was calculated as the percentage of viable cells at each concentration, in relation to the control cells. In addition, the half maximal inhibitory concentration (IC_50_) was calculated as the concentration required to inhibit the growth of tumor cells in culture by 50%, compared to the control cells.

Zymography analysis is considered a simple, sensitive, quantifiable, and functional assay for analyzing MMPs in condition media (21). Briefly, aliquots of conditioned media were subjected to electrophoresis in a gelatin-containing polyacrylamide gel, in the presence of sodium dodecyl sulfate (SDS) under non-reducing conditions, at 80 V for 3 h. After electrophoresis, the SDS gel was rinsed with distilled water, and again with a 2.5% solution of triton X100 twice, 30 min each time (renaturation), to remove the SDS. The gel slabs were then incubated at 37°C overnight in 0.1 M Tris HCL gelatinase activation buffer (pH 7.4) containing 10 mM CaCl_2_ with gentle agitation. Staining was performed with 0.5% Coomassie Brilliant Blue R250 (Sigma, MA), followed by intensive destaining. MMP-2 proteolysis areas appeared in the gels as clear bands against a blue background. Therefore, the surface and the intensity of the lysis bands, based on the grey levels, were compared in relation to the non-treated control wells and expressed as a percentage of the relative expression of gelatinolytic activity. The IC_50_ values were calculated as the concentration at which 50% of MMP inhibition occurred relative to the control cells.

### Cell apoptosis assay

To determine the possible apoptotic effect of Asn-AuNP conjugates, an apoptosis test was carried out using the ApoScreen Annexin V Apoptosis Kit (USA), which determines subpopulations of cells undergoing apoptosis by determining the phosphatidylserine (PS) molecules translocated from the inner leaflet of the phospholipid bilayer to the cell surface. Cells were preliminarily washed twice in cold PBS, and the PBS was removed from the cell pellet after the second wash. Cells were resuspended in cold buffer to the concentration of 1 × 10^6^ cells/mL. Then, 100 μL of cell suspension was added to each tube. About 10 μL of annexin V-R-PE was added to tube 2 and tube 4 of the Annexin V kit. Each tube was gently vortexed and then incubated for 15 min on ice, in dark condition, and 380 μL of cold buffer was added to each tube. Then, 10 μL of 7-AAD was added to tube 3 and tube 4. Quantification of the apoptotic cells was performed with a flow cytometry instrument (Partec PAS Germany). The results were expressed as a percentage of the apoptotic cells. Cells that had bound Annexin V-FITC (viable cells) showed green staining in the plasma membrane while those with no membrane integrity showed red staining throughout the nucleus and a halo of green staining on the cell surface.

### Statistical analysis

All Statistical analysis used with SPSS software (Chicago, IL, USA) and differences between different concentrations of the test groups (Asn-AuNP conjugates) and control groups (AuNPs) in cytotoxicity and zymography assay was compared with parametric t-test and one-way ANOVA. Statistical significance was designated as *P*-value<0.05.

## Results

### Biosynthesis of AuNPs and characterization

**Fig. 1:**
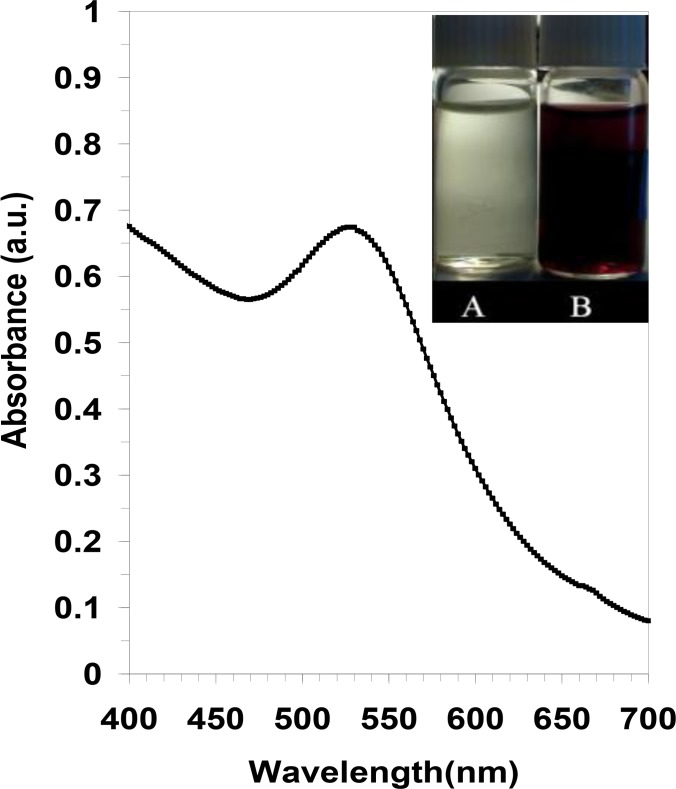
UV–visible spectrum of gold colloid was recorded after a period of 15 min: The inset shows a photograph of the solutions of chloroauric acid (1 mM) before (A) and after exposure to the free tannin ethanol extract of black tea (B).

Fig. 1 depicts the reaction of 1 mM chloroauric acid solution before (vessel A), and after incubation with tannin free ethanol extract of black tea for 15 min at room temperature(vessel B). The gold-containing solution (vessel A), which was a transparent yellow at first, turned into purple after the reaction was completed by the free tannin fraction of black tea ethanol extract (vessel B). The appearance of purple in the reaction vessel suggested the formation of AuNPs with a size <20 nm (22, 23). Subsequently, the prepared colloid was characterized with UV–visible spectroscopy. As illustrated in Fig. 1, a strong, absorption band with a maximum located at 524 nm was observed due to formation by the tannin free ethanol extract of black tea. This peak is assigned to a surface plasmon phenomenon well-documented for various metal nanoparticles with sizes ranging from 2 nm to 100 nm (22, 23).

**Fig. 2:**
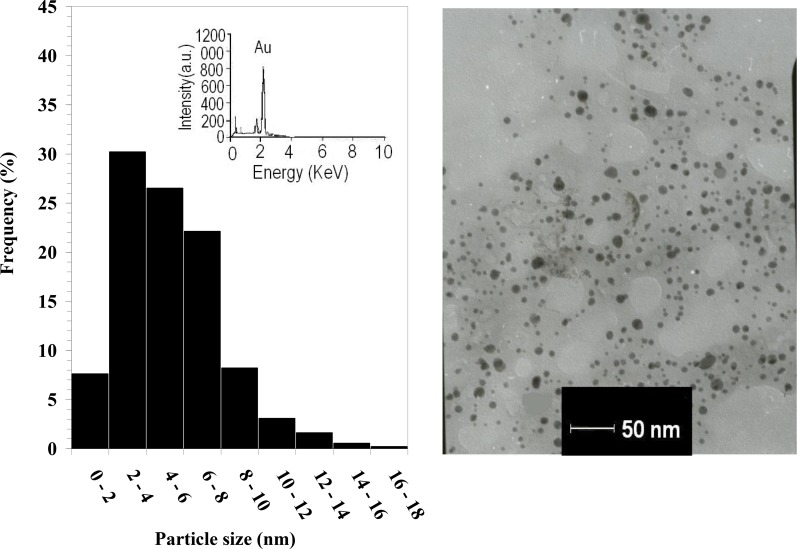
Transmission electron micrograph recorded from a small region of a drop-coated film of chloroauric acid solution treated with the free tannin ethanol extract of black tea (right-hand picture) for 15 min (scale bars correspond to 50 nm). The related particle size distribution histogram (left-hand picture) was obtained after 350 individual particles were counted for each sample. The inset in the left-hand illustration shows the energy-dispersive spectroscopy spectrum of the prepared gold nanoparticles. The gold X-ray emission peak is labeled. Strong signals from the atoms in the nanoparticles are observed in the spectrum and confirm the reduction of gold ions to gold nanoparticles.

The right-hand image in Fig. 2 shows the TEM image of the drop-coated film of the freshly prepared AuNPs, and the left-hand illustration in Fig. 2 demonstrates the particle size distribution histogram. The TEM image proves that the particles varied in size from 1.25 to 17.5 nm, and possessed an average size of 3 nm (Fig. 2). In the EDS analysis of the AuNPs, the presence of an elemental gold signal was confirmed in the sample (inset in the left-hand illustration in Fig. 2). The Au nanocrystallites display an optical absorption band peaking at 2.15 keV that is typical of the absorption of metallic gold nanocrystallites (24).

### Preparation of Asn-AuNP conjugates and their surface study

**Fig. 3:**
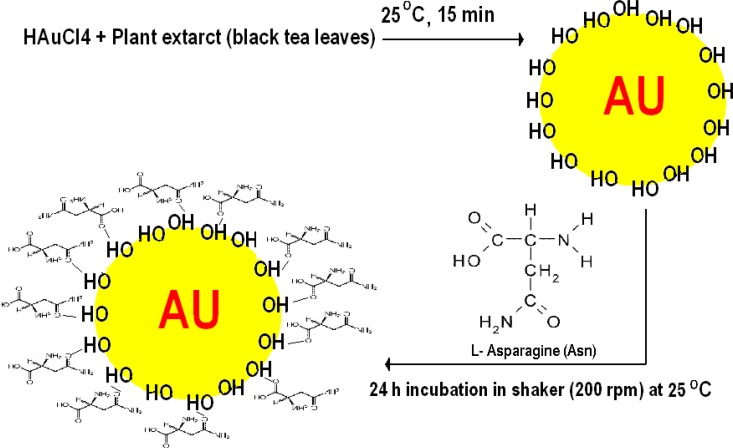
An artwork shows the procedure of synthesis of Asn-AuNPs. Hydroxyl surfaced gold nanoparticles were primarily fabricated by ethanol extract of black tea and subsequently were further coated by asparagine amino acid.

**Fig. 4:**
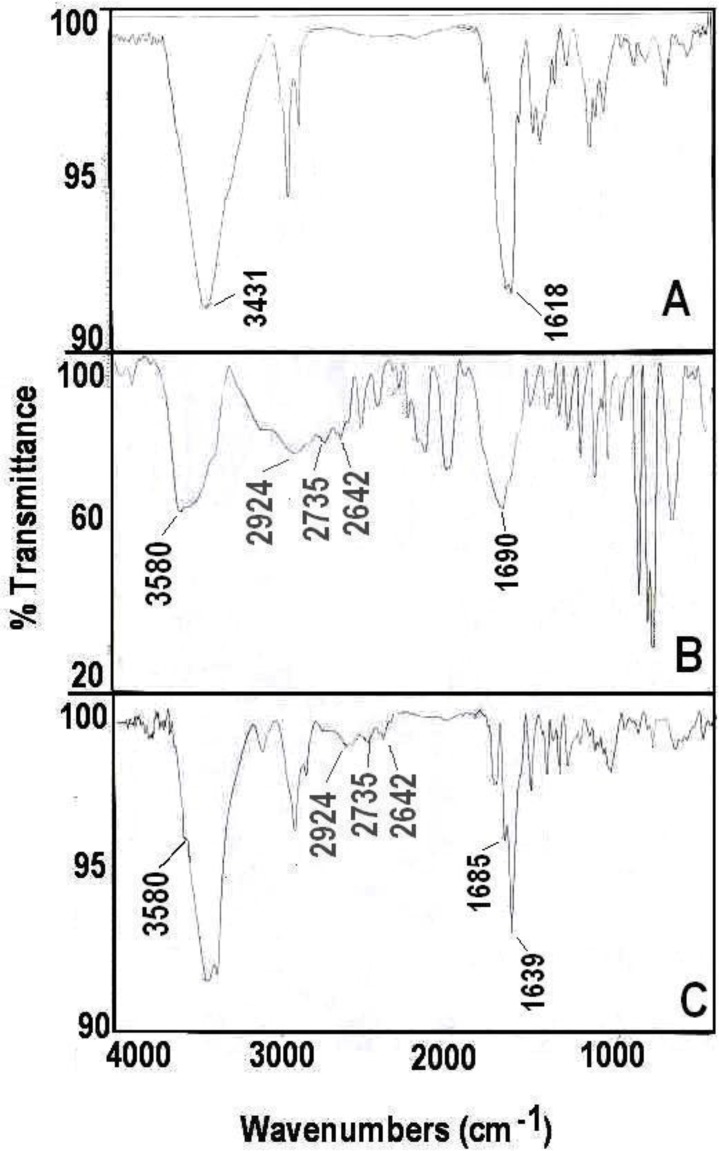
The Fourier transform infrared (FTIR) spectroscopy spectra of free Au NPs (A), asparagine powder (B), and Asn-AuNP conjugates (C)

The Asn-AuNP conjugates were prepared with the method described in the method section (Fig. 3), and their surface was investigated with FTIR spectroscopy and TGA assay. The FTIR spectra of asparagine free AuNPs, asparagine powder, and Asn-AuNP conjugates are shown in Fig. 4 (FTIR spectra A-C). Hydroxyl groups (3431 cm^−1^) on the surface of Au NPs prepared using ethanol extract of black tea (FTIR spectrum A). These hydroxyl groups on the surface of Au NPs may be derived from tannin molecules that not completely absorbed during refining process of ethanol extract of black tea leaves by Sephadex LH 20. The absorption bands, observed at 2640–3114 cm^−1^ in asparagine (FTIR spectrum B), were assigned to be the stretching vibration band of free OH in the carboxylic acid group. In addition, the N-H vibration band in asparagine is due to N-H bonding at 3580 (FTIR spectrum B). All mentioned absorption bands contributed to asparagine being observed in the FTIR spectrum (C) of the washed Asn-AuNP conjugates, indicating the existence of this amino acid on the surface of the conjugated AuNPs.

Moreover, FTIR spectroscopic examination revealed that asparagine conjugated onto hydroxyl capped AuNps via hydrogen bonding with carbonyl group of asparagine (FTIR spectra A, C). The absorption band, observed at 1690 cm^−1^ in asparagine (spectrum B), were assigned to be CO, while in an Asn-AuNP conjugates this band was shifted to 1639 cm^−1^ (spectrum C). In addition, un-bonded carbonyl groups (1685 cm^−1^) can be also detected on the surface of Asn-AuNP conjugates.

**Fig. 5:**
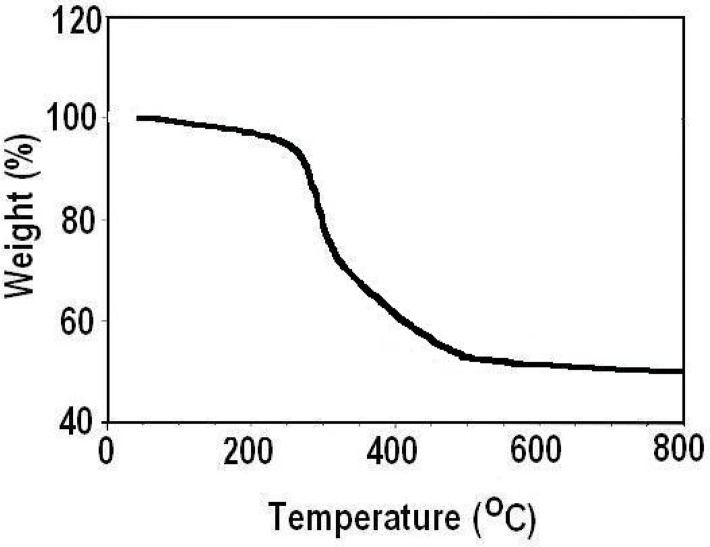
Weight loss of Asn-AuNP conjugates as a function of temperature, determined by the thermogravimetric method

Fig. 5 shows the weight loss of the Asn-AuNP conjugates as a function of temperature, determined by the thermogravimetric method. The weight loss in the TGA curve indicated carbon oxidation, thereby proving the presence of organic coating in the sample and confirmed that the Asn-AuNP conjugates were capped with organic materials.

### MTT assay

**Fig. 6:**
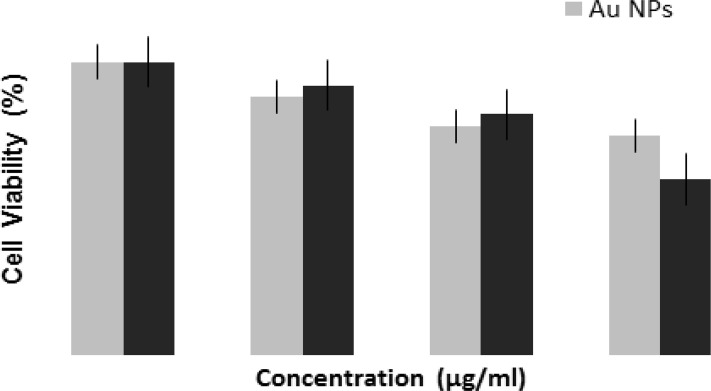
The dose-dependent response of CCRF-CEM cells after treatment with AuNPs and Asn-AuNP conjugates. A *P*-value of <0.05 was considered statistically significant*

The percentage of inhibition values was calculated as 100– [(Optical density_test sample_ / Optical density_blank sample_) × 100]. The percentage inhibition curves were obtained by plotting the percentage of inhibition against the concentration of the Asn-AuNP conjugates and free AuNPs. Fig. 6 shows the dose-dependent response of the CCRF-CEM cells after treatment with AuNPs and Asn-AuNP conjugates. According to the bar chart, the antiproliferative effect of AuNPs increases when used in combination with asparagine molecules at 39 °C. At the concentration of 300 μg/mL, the percentage of viable cells significantly decreased to 74% in the AuNPs group, while that of the Asn-AuNP conjugates decreased to 60% in the same slight hyperthermia condition (39 °C).

### Zymography analysis and apoptosis assay

**Fig. 7:**
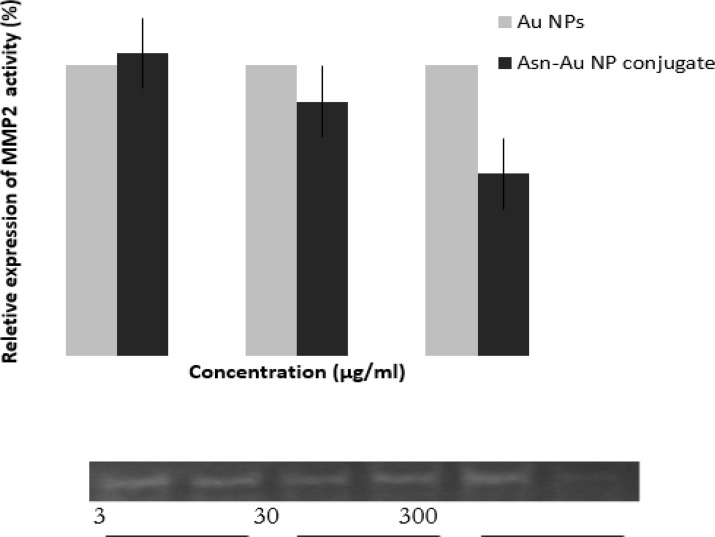
Zymography results after treatment with 3, 30, and 300 μg/mL of Asn-AuNP conjugates and free AuNP. Upper panel: Bar Diagram Depiction, Lower Panel: Electrophoresed Zymogram Gel Picture. At the concentration of 300 μg/mL, the level of MMP expression significantly decreased when the cells were treated with Asn-AuNP conjugates, rather than non-coated AuNPs

**Fig. 8:**
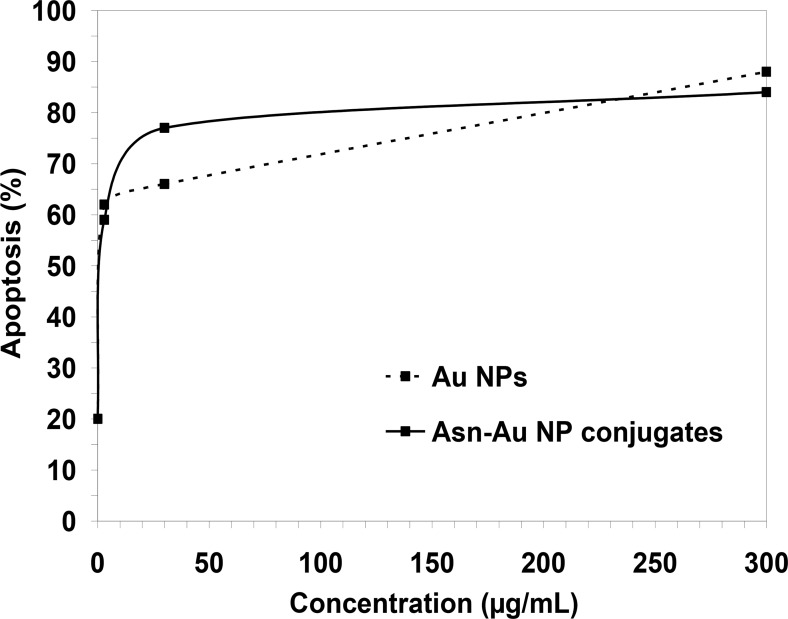
The apoptosis results after treatment with 3, 30, and 300 μg/mL of Asn-AuNP conjugates and free AuNPs. The level of apoptosis increased in both groups in almost similar patterns.

Fig. 7 shows the zymography results after treatment with 3, 30, and 300 μg/mL of Asn-AuNP conjugates and AuNPs. As is clear from the graph, at the concentration of 300 μg/mL, the level of MMP expression significantly decreased when the cells were treated with Asn-AuNP conjugates, rather than non-coated AuNPs. Fig. 8 demonstrates the apoptosis that occurred after treatment with Asn-AuNP conjugates and AuNPs. According to Fig. 8, in the absence of gold nanomaterials, the increase in temperature caused 20% apoptosis in cells. In cells treated with AuNPs, the level of apoptosis increased sharply until the concentration of 30 μg/mL, where 66% of cells underwent apoptosis. At the highest concentration of 300 μg/mL, the percentage of apoptosis increased to 88% in the presence of AuNPs and 84% in the presence of Asn-AuNP conjugates. Taken together, the level of apoptosis increased in both groups in almost similar patterns. However, the Asn-AuNPs showed a higher apoptosis effect at a lower concentration (30 μg/mL).

**Fig. 9:**
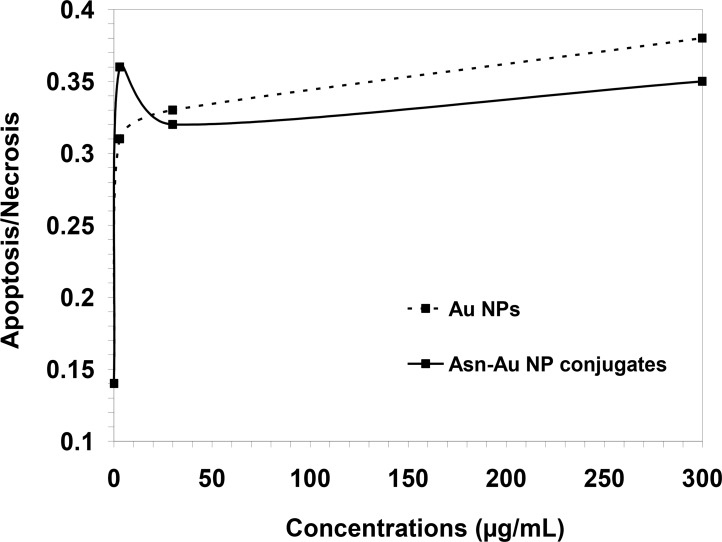
The ratio of apoptosis/necrosis in cells treated with AuNPs and Asn-AuNP conjugates

Fig. 9 shows the ratio of apoptosis/necrosis in cells treated with AuNPs and Asn-AuNP conjugates. In the presence of AuNPs, the ratio of apoptosis/necrosis increased from 0.31 at the concentration of 3 μg/mL to 0.38 at the concentration of 300 μg/mL. However, in the presence of Asn-AuNP conjugates, this ratio increased sharply to 0.36 at the concentration of 3 μg/mL and then significantly decreased to 0.32 at 30 μg/mL.

The ratio of apoptosis/necrosis remained below 0.36 as the concentration increased.

The percentage of viable cells was significant in the Asn-AuNP group compared to the AuNPs group (*P*-value less than 0.05). Moreover, at the concentration of 300 μg/mL, the percentage of viable cells in the Asn-AuNP group decreased to 60%, which was statistically significant.

## Discussion

Tumor cells selectively intake asparagine as a necessary amino acid (14). Therefore, if asparagine molecules may have enhanced cytotoxic and apoptotic properties of gold nanoparticle (AuNPs), compared to non-coated AuNPs. The apoptotic effect of AuNPs in B-chronic lymphocytic leukemia cells increases when the AuNPs are coated with anti-VEGF antibodies (16). Both PAMAM-coated and citrate-coated were demonstrated AuNps induce cytotoxicity in HepG2 cells or PBMC. The immune system cells (PBMC) were less sensitive to DNA damage than cancer HepG2 cells, upon exposure to AuNps (25). Gold nanomaterials are able to form strong bonds particularly with thiol, hydroxyl and amine groups within organic molecules (26, 27). Therefore, in this study, asparagine molecules were attached to hydroxyl capped AuNPs through the carbonyl group within the asparagine molecule (Fig. 3) (Scheme 1).

The results of this work revealed that at 39 °C, Asn-AuNP conjugates significantly inhibited MMPs, compared to non-asparagine coated AuNPs. In terms of cytotoxicity, a remarkable decrease was observed in the percentage of viable cells treated with Asn-AuNP conjugates, rather than asparagine free AuNPs. Moreover, the AuNPs and Asn-AuNP conjugates enhanced the level of apoptosis at almost similar rates, and no significant difference was observed in the level of apoptosis caused by gold nanomaterials.

Fig. 8 indicates that the percentage of apoptotic cells increased from 20% to around 60% when cells were treated with only 3 μg/mL of either AuNPs or Asn-AuNP conjugates. As clear from Fig. 9, in cells treated with higher concentrations of Asn-AuNP conjugates, the ratio of apoptosis/necrosis is lower than that of AuNPs, which means that the presence of asparagine on the surface of nanoparticles increased the level of necrosis among the cells. A possible explanation may be that having bipolar properties and high solubility in cell membranes, Asn-AuNP conjugates may have entered into cells more efficiently than non-asparagine coated AuNPs, as other researchers previously shown (28). Therefore, it sounds plausible that, at higher concentrations, the intake of Asn-AuNP conjugates into cells increased, so that necrosis dominated apoptosis and the apoptosis/necrosis ratio decreased in cells treated with Asn-AuNP conjugates, compared to those who received free, none-asparagine coated AuNPs. Because of the increase in temperature, Asn-AuNP conjugates may have entered the cells more effectively. Hence, as the level of Asn-AuNP conjugates increased in the medium, their uptake into cells increased, which consequently induced more necrosis and less apoptosis.

Furthermore, the effect of the temperature should not be ignored. Cells can normally tolerate 39 °C temperature by releasing heat-shock proteins (HSPs) that allow the cells to maintain homeostasis at temperatures below 40 °C (29). Cells begin to show signs of apoptosis when heated to temperatures between 41 and 47 °C while increasing temperatures above 50 °C will cause less apoptosis and more necrosis among cells (30). However, the results of this work suggest that Asn-AuNP conjugates and AuNPs both enhanced apoptosis and necrosis at 39 °C temperature, and even more frank necrosis (rather than apoptosis) occurred when the cells were treated with higher concentrations of Asn-AuNP conjugates. Therefore, Asn-AuNP conjugates may interfere with the function of HSPs, which literally reduced the thermotolerance of viable cells. More experiments must be conducted to clarify the molecular interactions of Asn-AuNP conjugates within cells.

## Conclusion

The use of gold nanoparticles to treat cancer cells is growing and continues to expand rapidly. The apoptotic and cytotoxic effects of AuNPs are well known, and so far, a number of protocols have been devised to enhance the anti-cancer profile of AuNPs. We suggested that when hydroxyl capped biogenic AuNPs are coated with asparagine molecules and the temperature is slightly increased by 2 °C, the apoptosis is not only enhanced among cells but also shifts to necrosis in higher concentrations of Asn-AuNP conjugates. More investigations should be carried out to explain the exact mechanism underlying the necrotic effects of Asn-AuNPs.

## Ethical considerations

Ethical issues (Including plagiarism, informed consent, misconduct, data fabrication and/or falsification, double publication and/or submission, redundancy, etc.) have been completely observed by the authors.
